# MicroRNA signature in classical Hodgkin lymphoma

**DOI:** 10.1007/s13353-021-00614-7

**Published:** 2021-02-05

**Authors:** Julia Paczkowska, Maciej Giefing

**Affiliations:** Institute of Human Genetics, Polish Academy of Sciences, Strzeszynska 32, 60-479 Poznan, Poland

**Keywords:** cHL, HRS, Epigenetics, microRNA

## Abstract

Classical Hodgkin lymphoma (cHL) is one of the most prevalent lymphomas with a unique cell composition compared to other lymphoma entities. Rare, malignant Hodgkin and Reed-Sternberg (HRS) cells embedded with an extensive but ineffective immune infiltration were previously characterized by a large number of genetic and epigenetic alterations. Recently, microRNA profiling studies highlighted the importance of small non-coding RNA in cHL. This review summarizes available literature data and provides a detailed comparison of four studies where cHL cell lines and microdissected HRS cells were used. Several microRNAs were found to be consistently up- (let-7-f, mir-9, mir-21, mir-23a, mir-27a, mir-155, and mir-196a) or downregulated (mir-138 and mir-150) in cHL. These deregulated microRNAs are involved in the processes crucial for cHL pathogenesis, such as impaired B cell development (mir-9, mir-150, and mir-155), NFκB hyperactivation (mir-155 and mir-196a), and immune evasion (mir-138). Therefore, the deregulation of microRNA expression can be considered a complementary mechanism to genetic alterations promoting lymphomagenesis. Moreover, the expression of let-7f, mir-9 and mir-27a is specific for cHL and can serve as a biomarker to distinguish this lymphoma from other B cell lymphomas. However, additional in-depth and high throughput analysis of microRNA expression in HRS cells is necessary to decipher the complete picture of microRNA in cHL.

**Supplementary Information:**

The online version contains supplementary material available at 10.1007/s13353-021-00614-7.

## Introduction

Given the unique cell composition of the tumor microenvironment, with only a small number of malignant Hodgkin and Reed-Sternberg (HRS) cells (~ 1% of tumor volume) embedded with an extensive immune infiltration, genetic and epigenetic studies in classical Hodgkin lymphoma (cHL) become laborious (Mathas et al. 2016). Despite those difficulties, several groups have undertaken the challenge of characterizing the microRNA expression profile of cHL. As a consequence of different experimental approaches and type of samples used for these analyses, many inconsistencies can be found between the studies. Therefore, with this review, we intend to provide a comprehensive summary of what has already been discovered in the field and describe the function of microRNAs, which were found to be consistently deregulated in cHL in the previous studies. In addition, we propose how these new players fit into the main processes of cHL pathogenesis like impaired B cell development, NFĸB hyperactivation, or immune evasion.

## MicroRNA profiles in cHL

**Fig. 1:**
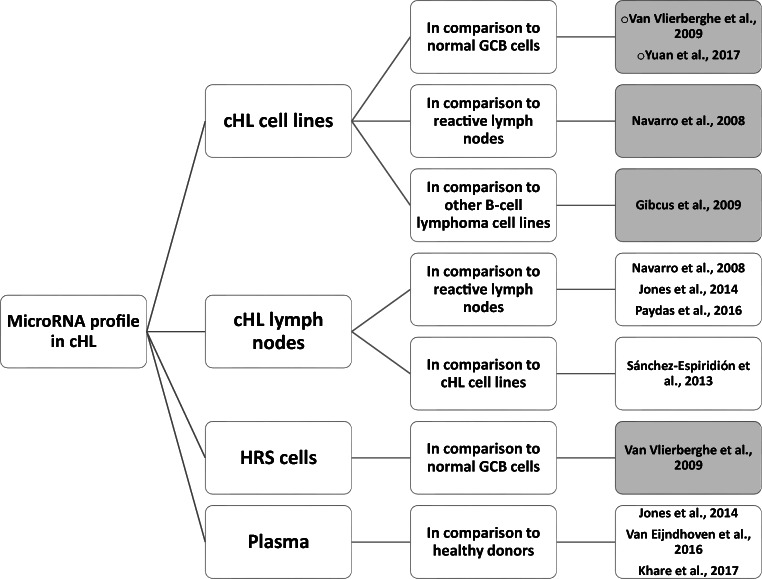
Summary of previously published microRNA profiling studies in cHL. Studies selected for detailed comparison are labeled in gray

In this review, published microRNA studies of cHL were divided into four groups, regarding differences in the experimental questions (Fig. 1). In the first, the authors used cHL cell lines and compared the results to normal GCB cells (Van Vlierberghe et al. 2009; Yuan et al. 2017), reactive lymph nodes (RLNs) (Navarro et al. 2008), or other B cell lymphoma cell lines (Gibcus et al. 2009). In the second group, the authors used whole lymph nodes (LNs) from cHL cases to establish microRNA profiles, and these were analyzed either in relation to RLNs (Navarro et al. 2008; Jones et al. 2014; Paydas et al. 2016) or cHL cell lines (Sánchez-Espiridión et al. 2013). Taking into account the scarcity of HRS cells in the tumor microenvironment, analysis of microRNA expressions in the whole lymph node tissues allows the detection of only highly overexpressed microRNAs. In the third group, microdissected HRS cells were compared to normal GCB cells (Van Vlierberghe et al. 2009). This approach is probably the most accurate way to address the question of microRNA deregulation in the consequence of lymphomagenesis. Despite the excellent study design, only a small number of microRNAs were evaluated in this study. In the fourth group, to translate the increasing knowledge about microRNAs into clinical practice, plasma samples from cHL patients were used (Jones et al. 2014; Van Eijndhoven et al. 2016; Khare et al. 2017). MicroRNAs in plasma can serve as biomarkers and prediction factors. However, their signature in the periphery can differ from HRS cells and may not reflect malignant cells’ biology in this disease.

To the best of our knowledge, this is the second article that reviews microRNA profiles in cHL after the manuscript published in 2017 by Cordeiro et al. 2017 Our manuscript’s significant advantage is the inclusion of the newest high throughput data obtained in the last years.

## MicroRNAs deregulated in cHL

**Fig. 2:**
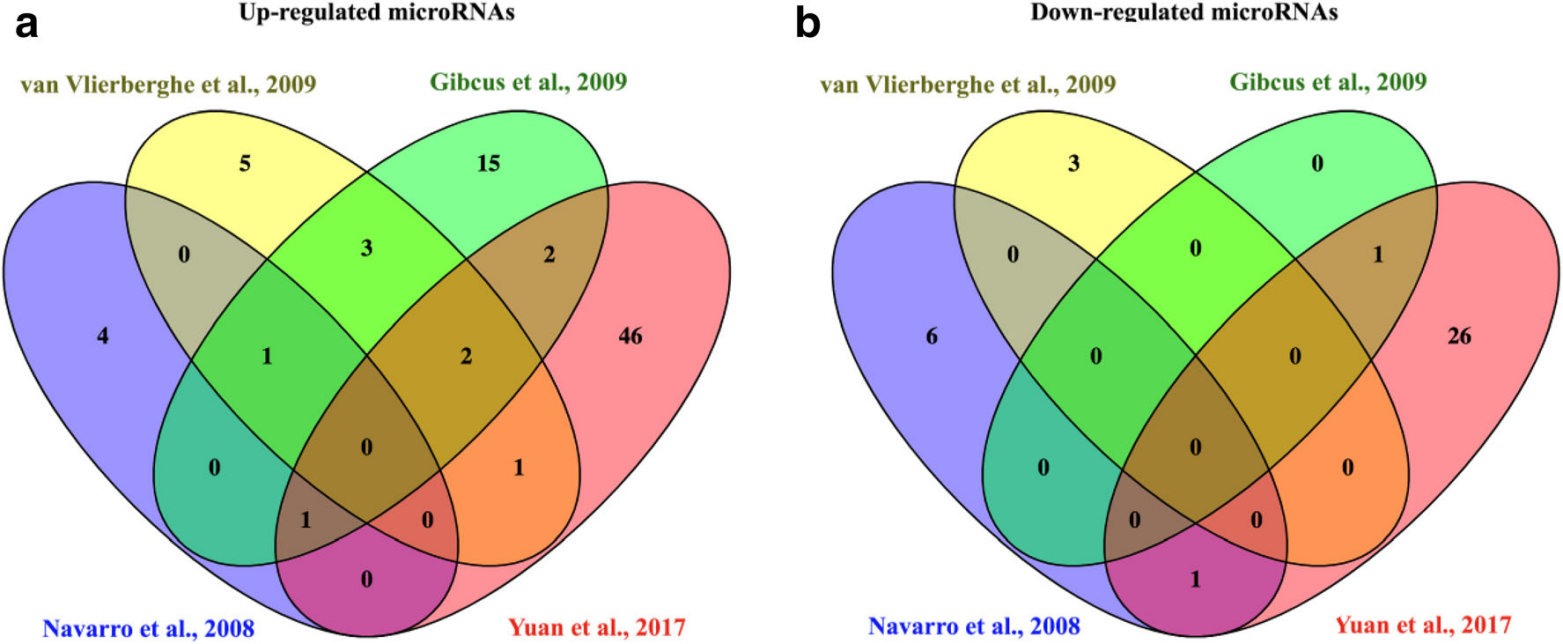
Venn diagram (Oliveros 2007) showing the overlap between four previous miRNA profiling studies in cHL (Navarro et al. 2008; Van Vlierberghe et al. 2009; Gibcus et al. 2009; Yuan et al. 2017). For detailed information, see Supplementary Table 2

After careful analysis of all previously published results, we have selected four publications for a more detailed comparison. The main criteria of the selection was the type of samples used for microRNA profiling. We have chosen all studies performed in cHL cell lines and microdissected HRS cells. It allows to evaluate the microRNA profiles in a broad context with different controls: normal GCB cells, RLNs, and non-Hodgkin lymphoma (NHL) cell lines. Finally, we have compared the publication by Yuan et al., where high throughput small RNA NGS sequencing was used, with three earlier reports (Navarro et al. 2008; Van Vlierberghe et al. 2009; Gibcus et al. 2009). By this comparison, we have identified six microRNAs (let-7-f, mir-9, mir-23a, mir-27a, mir-155, and mir-196a) upregulated and two microRNAs downregulated (mir-138 and mir-150) in cHL based on the overlap between the data presented by Yuan et al. and at least one of the three previous studies. We have also found mir-21 with a known oncogenic role that was shown upregulated in three of the four analyzed studies (Navarro et al. 2008; Sánchez-Espiridión et al. 2013; Van Eijndhoven et al. 2016) (Table 1, Supplementary Table 1 and Fig. 2).

**Table 1:** Comparison between four selected microRNA profiling studies

RT-qPCR						small RNA-seq	
Navarro et al. 2008		Van Vlierberghe et al. 2009		Gibcus et al. 2009		Yuan et al. 2017	
Deregulated miRNAs in 3 cHL cell lines in comparison to 10 RLNs		Deregulated miRNAs in 9 microdissected HRS pools and 4 cHL cell lines in comparison 3 GCB CD77^+^ pools		Deregulated miRNAs in 5 cHL cell lines in comparison to 15 NHL cell lines		Deregulated miRNAs in 4 cHL cell lines in comparison to 3 GCB pools	
UP	DOWN	UP	DOWN	UP	DOWN	UP	DOWN
mir-21	mir-23b	mir-9	mir-200a#	let-7-e	**mir-150**	let-7a-3p	mir-2_4723-5p
mir-27a	mir-205	mir-16	mir-520a#	let-7-f		mir-let-7b-3p	mir-7_18763-5p
mir-147	mir-126	mir-18a#	mir-614	mir-9		let-7b-5p	mir-17_35723-3p
mir-182	mir-135a	mir-20a		mir-15b		let-7-f-2-3p	mir-20b-5p
mir-183	**mir-138**	mir-21		mir-16		mir-9-3p	mir-28-3p
mir-216	mir-204	mir-30a-5p		mir-19a		mir-9-5p	mir-28-5p
	mir-335	mir-30b		mir-19b		mir-12_28421-5p	mir-30a-3p
		mir-140		mir-20a		mir-23a-3p	mir-30a-5p
		mir-155		mir-21		mir-23a-5p	mir-141-3p
		mir-186		mir-23a		mir-24-3p	mir-143-3p
		mir-196a		mir-25		mir24–2-5p	mir-148a-3p
		mir-374		mir-27a		mir-27a-3p	mir-148a-5p
				mir-29a		mir-27a-5p	mir-130a-3p
				mir-29c		mir-33b-5p	mir-138-1-3p
				mir-30b		mir-92b-3p	**mir-138-5p**
				mir-92		mir-98-3p	**mir-150-3p**
				mir-93		mir-99a-5p	**mir-150-5p**
				mir-106a		mir-101-5p	mir-181a-2-3p
				mir-106b		mir-147b	mir-223-3p
				mir-125a		mir-149-5p	mir-340-5p
				mir-130b		mir-153-3p	mir-363-3p
				mir-142-3p		mir-155	mir-548 h-5p
				mir-155		mir-190a-5p	mir-577
				mir-191		mi-195-5p	mir-598-3p
						mir-196-5p	mir-2116-3p
						mir-296-5p	mi-3150b-3p
						mir-301b-3p	mir-3681-5p
						mir-320a	mir-5100
						mir-330-3p	mir-6500-3p
						mir-330-5p	
						mir-345-5p	
						mir-378a-3p	
						mir-424-3p	
						mir-424-5p	
						mir-450a-5p	
						mir-450b-5p	
						mir-497-5p	
						mir-542-3p	
						mir-550a-3p	
						mir-550a-5p	
						mir-550a-3-5p	
						mir-615-3p	
						mir-625-5p	
						mir-744-3p	
						mir-760	
						mir-877-5p	
						mir-944	
						mir-1254	
						mir-1276	
						mir-1301-3p	
						mir-3176	
						mir-3200-3p	
						mir-4461	
						mir-5701	
						mir-7705	

Upregulated microRNAs which were identified by Yuan et al. and found also in at least one of three previous studies (Navarro et al., van Vlierberghe et al., Gibcus et al.) are underlined, and downregulated microRNAs are marked in bold. Moreover, mir-21 found overexpressed in the three previous studies (Navarro et al., van Vlierberghe et al., Gibcus et al.) is underlined

## MicroRNAs upregulated in cHL

### let-7f-2 (MI0000068)

Upregulation of let-7f-2 was described in cHL cell lines in comparison to normal GCB cells and other B cell derived lymphoma cell lines (Gibcus et al. 2009; Yuan et al. 2017). No additional functional studies were performed to illustrate the role of this microRNA in cHL. Further investigation is necessary to understand the function of let-7f-2 in lymphoma.

### mir-9 (MI0000466)

Mir-9 was found upregulated by Navarro et al. in FFPE sections of lymph nodes from cHL patients in comparison to RLNs (Navarro et al. 2008). Similar observations were done in cHL cell lines and microdissected HRS cells independently by three groups (Van Vlierberghe et al. 2009; Gibcus et al. 2009; Yuan et al. 2017). It proved that the overexpression of mir-9 in cHL is not only limited to the whole lymph node sections and cHL cell lines, but what important, is also present in malignant cells from cHL patients. Moreover, mir-9 overexpression is a unique characteristic of cHL compared to other B cell derived cell lines (Gibcus et al. 2009).

The importance of the CD99-mir-9-PRDM1 regulatory axis in cHL was previously described by Huang et al. (Huang et al. 2012). The authors showed recurrent downregulation or loss of these two master regulators (*CD99* and *PRDM1*) of terminal B cell differentiation into plasma cells in the majority of the cHL cell lines and HRS cells. They proposed the overexpressed mir-9 to be at least partially responsible for the downregulation of these genes. The authors refer to the previous study by Nie et al. where direct interaction between mir-9 and *PRDM1* was demonstrated in a luciferase reporter assay and the correlation between *PRDM1* and mir-9 expressions was observed (Nie et al. 2008). To further validate this interaction Huang et al. performed mir-9 inhibition experiments, which in line with the previous study resulted in PRDM1 overexpression. These experiments validated that PRDM1 loss in cHL may be explained by the high expression of mir-9 in this lymphoma and may contribute to the aborted differentiation of HRS cells towards terminal B cells (Huang et al. 2012).

The significance of overexpressed mir-9 in cHL is also related to its direct interaction with *DICER1* and *ELAVL1* (HuR) as demonstrated in three cHL cell lines (L-428, L-540, and KM-H2). Interestingly, *ELAVL1* (HuR) was shown to modulate the expression of IL-5, IL-6, CCL-5, and TNF-alpha cytokines harboring the HuR-binding motif, which in consequence can influence the cross-talk between HRS cells and the immune infiltration in the tumor microenvironment. In conclusion, mir-9 can indirectly regulate several cytokines expression in *ELAVL1* (HuR) dependent manner. In line with this observation, in vivo experiment showed that inhibition of mir-9 decreases tumor growth and increases the number of DICER1 and ELAVL1 (HuR) positive cells (Leucci et al. 2012).

### mir-23a (MI0000079)

Overexpression of mir-23a in cHL cell lines was reported by Gibcus et al. (2009) and Yuan et al. (2017) in comparison to both other B cell lymphoma cell lines and nonmalignant GCB cells, respectively. Mir-23a was also described as upregulated in FFPE lymph nodes from cHL patients compared to reactive lymph nodes (Navarro et al. 2008). Surprisingly, as reported by Yuan et al., mir-23a inhibition by miRZIP-23a-3p in cHL cell lines had no adverse effect on cell proliferation/viability or resulted even in an increase of cell viability as observed in the GFP competition assay. No further validation of mir-23a function was presented; therefore, its detailed role in cHL remains unclear. Mir-23a overexpression is not exclusively present in cHL but was also described in DLBCL patients as compared to RLNs from formalin-fixed, paraffin-embedded tissues (Wang et al. 2014). Based on this study, mir-23a might be used as a prognostic factor in DLBCL because high expression level of mir-23a was significantly associated with poor overall survival. However, the mechanism of mir-23a action is also unknown in DLBCL.

### mir-27a (MI0000085)

Mir-27a was shown to be upregulated in cHL in three previously published studies (Navarro et al. 2008; Gibcus et al. 2009; Yuan et al. 2017). Mir-27a was described as upregulated in FFPE lymph nodes from cHL patients compared to reactive lymph nodes (Navarro et al. 2008). This microRNA emerges as a cHL-specific overexpressed microRNA and allows the discrimination of cHL cell lines from normal GCB cells and other B cell malignancies.

Until now, only Yuan et al. characterized the role of mir-27a in cHL. The authors showed a significant decrease in the number of GFP-positive cells after miRZip based silencing of miR-27a-3p in two (L-540 and L-1236) of four evaluated cell lines. Detailed effects of mir-27a-3p inhibition were not further investigated, and therefore the cohort and function of the putative target genes for mir-27a remain to be elucidated. However, a recent report suggests mir-27a oncogenic involvement in breast cancer, and the authors showed the direct interaction between mir-27a and the tumor suppressor *PTEN* gene and the pro-apoptotic *BAX* gene in luciferase assay (Wu et al. 2018). Moreover, overexpression of this microRNA led to the downregulation of both genes on the protein level in breast cancer cell lines.

### mir-155 (MI0000681)

Mir-155 overexpression was postulated by three groups and described in microdissected HRS cells and cHL cell lines (Van Vlierberghe et al. 2009; Gibcus et al. 2009; Yuan et al. 2017). In 2005, microRNA deregulation in cHL was reported for the first time with mir-155 overexpression shown as an example. Moreover, mir-155 was postulated to be overexpressed in cHL by three other independent groups (Sánchez-Espiridión et al. 2013; Paydas et al. 2016; Van Eijndhoven et al. 2016). In the first study by Kluiver, mir-155 expression was found to be characteristic for both cell lines and primary cHL cases (Kluiver et al. 2005). The non-coding *BIC* (MIR155HG) gene with which mir-155 is transcribed is also upregulated in PMBL and DLBCL, with a higher proportion of *BIC* positive cells in ABC-like DLBCL that shares several similarities with cHL, than in GCB-like DLBCL’s subtype. Regarding the function of mir-155, it was shown to control the GC reaction and to promote lymphomagenesis by negative regulation of *GCSAM* (*HGAL*) expression, a specific germinal center gene that under physiological conditions decreases cell motility (Jiang et al. 2010). Consequently, mir-155 shows the potential to increase malignant lymphocytes’ motility by direct interaction with *GCSAM* (*HGAL*) (Dagan et al. 2012).

The oncogenic role of mir-155 in lymphomas development was well documented by Babar et al. who showed, using a mouse model, that overexpression of mir-155 in lymphoid tissues causes disseminated lymphoma, whereas inhibition of this microRNA leads to tumor regression in mice model after lymphoma development (Babar et al. 2012). Moreover, Gibcus et al. experimentally proved the direct interaction between the cHL overexpressed mir-155 and *IKBKE* (inhibitor of NFκB kinase subunit epsilon) in luciferase reporter assays suggesting its putative contribution to NFĸB constitutive activation in HRS cells (Gibcus et al. 2009).

### mir-196a (MI0000238)

Mir-196a was reported as upregulated in microdissected HRS cells and cHL cell lines in two studies (Van Vlierberghe et al. 2009; Yuan et al. 2017). However, the exact role of this microRNA in the pathogenesis of cHL or other B cell lymphomas was not further discussed.

In contrast to lymphomas, mir-196a involvement was previously described in solid tumors. Mir-196a was found to act as an oncomiR by promoting tumor growth and metastasis in breast cancer (Jiang et al. 2018) and progression of hepatocellular carcinoma (Wang et al. 2019). These oncogenic effects have been supported by functional analysis where direct target genes for mir-196a like *IκBα*, *SPRED1*, and *RUNX2* were experimentally characterized. In the context of cHL biology, especially the direct interaction and repression of *IκBα* (inhibitor of NFκB) by overexpressed mir-196a seems crucial as it might contribute to the constitutive hyperactivation of the NFκB pathway in HRS cells (Wang et al. 2019).

### Mir-21 (MI0000077)

Mir-21 was reported as upregulated in three of four studies analyzed in this review (Navarro et al. 2008; Van Vlierberghe et al. 2009; Gibcus et al. 2009). Although it was not described as significantly altered in the study by Yuan et al., its expression was 3.4 times higher in cHL cell lines than in GCB cells (avg reads in HL, 136,903; avg. reads in GCB, 40,217), and it was the most abundant microRNA in cHL cell lines (Yuan et al. 2017). Mir-21 was also found overexpressed in the studies by Navarro et al. (2008), Sánchez-Espiridión et al. (2013), and Van Eijndhoven et al. (2016).

The oncogenic function of mir-21 in cHL is well documented (Yuan et al. 2018). Using the GFP competition assay, the authors demonstrated that miRZip-based inhibition of mir-21 significantly decreases the percentage of GFP-positive cells in cHL cell lines. Moreover, mir-21 inhibition decreases cell growth and induces apoptosis in cHL cell lines. These effects can be at least partially explained by the direct interaction of mir-21 with its newly identified targets *BTG2* and *PELI1*.

## MicroRNAs downregulated in cHL

### mir-138 (MI0000476)

In contrast to the larger group of cHL upregulated microRNAs, only two were found to be downregulated, namely, mir-138 and mir-150, in at least two of the discussed studies. Mir-138 downregulation has been described by Navarro et al. as a result of the analysis of FFPE lymph node sections from cHL patients and cHL cell lines and further confirmed by Yuan et al. Neither functional nor clinical relevance of this deregulation was investigated in cHL. However, the potential importance of this microRNA in cHL lymphomas comes from its direct interaction with 3′UTR of *PD-L1* demonstrated using the luciferase reporter assay in colorectal cancer cell lines (Zhao et al. 2016). Overexpression of PD-L1 in both HRS cells and nonmalignant tumor-associated macrophages leads to exhaustion of infiltrating immune cells, previously described in cHL (Carey et al. 2017). Importantly, anti-PD-1 treatment (Nivolumab) was found to be especially effective in relapsed/refractory cHL, showing the significant role of the PD-1/PD-L1 pathway in this malignancy. According to Zhao et al., functional restoration of the downregulated mir-138, for example, by using mir-138 mimic, can be considered a novel, additional therapeutic strategy to target the PD-1/PD-L1 axis in cHL and other cancers (Zhao et al. 2016).

### mir-150 (MI0000479)

Mir-150 is the second downregulated microRNA in cHL described by both Gibcus et al. and Yuan et al. In these two studies, mir-150 was downregulated in cHL cell lines compared to NHL cell lines and normal GCB cells. It was previously shown that mir-150 is involved in normal hematopoiesis by controlling B and T cell differentiation (Vasilatou et al. 2010).

Several previous studies shed light on its role in other B cell malignancies. Mir-150 was shown to drastically reduce the growth of Burkitt lymphoma cell lines by the MYC-miR-150-*ZDHHC11/B*-MYB network. In this complex pathway, high MYC level downregulates mir-150 expression to protect the oncogenic and lymphoma related MYB from mir-150-mediated repression. MYC-driven mir-150 downregulation results from the induced expression of *ZDHHC11B*, a target for mir-150, which acts as an endogenous sponge for this microRNA. Importantly, the authors extend their findings on MYC, *ZDHHC11/B*, MYB upregulation, and mir-150 downregulation also to other lymphoma entities including cHL (Dzikiewicz-Krawczyk et al. 2017). In line with these results, in the published mRNA expression profiles, *MYB* was found strongly overexpressed in cHL cell lines in comparison to normal B cells at different stages of differentiation (Küppers et al. 2003). This together suggests an important role of mir-150 in controlling the oncogenic MYB, a function impaired in cHL and other lymphomas.

## Conclusions

In this review, we have summarized the available data on microRNAs deregulated in cHL. Since 2008, several studies were performed, and many different approaches were applied to understand the role of microRNAs in this disease. Despite the obvious technical differences between the studies, we have found several microRNAs that are consistently up- (let-7-f, mir-9, mir-21, mir-23a, mir-27a, mir-155, and mir-196a) or downregulated (mir-138 and mir-150) in cHL.

Some of the deregulated microRNAs are characteristic markers expressed exclusively in cHL that distinguish this lymphoma from other lymphoma entities. These include let-7f, mir-9, and mir-27a (Gibcus et al. 2009). Importantly, the cohort of described microRNAs is also directly involved in cHL pathogenesis by regulating many important genes or entire signaling pathways. Based on the reviewed data, an essential role of these microRNAs emerges in several crucial processes in cHL pathogenesis.

(I) Impaired B cell development. Overexpression of oncogenic mir-9 and mir-155 contributes to impaired B cell development and GC reaction by targeting *PRDM1* and *HGAL*, respectively (Nie et al. 2008; Jiang et al. 2010). In consequence, further differentiation of the centrocytes into plasma cells is blocked. In addition, mir-150, which plays a significant role in normal hematopoiesis by controlling B and T cell differentiation, is lost in cHL and has a suppressive role in this disease (Vasilatou et al. 2010; Dzikiewicz-Krawczyk et al. 2017).
(II) NFκB hyperactivity. Strong and constitutive NFκB activation is a hallmark of cHL and essential for HRS cells’ survival (Küppers 2012). In this context, the described overexpression of oncogenic mir-196a promotes NFκB activation by repressing IκBα, the canonical pathway inhibitor (Wang et al. 2019). Moreover mir-155, in extent to its function in B cell development, can directly target *IKBKE*—another inhibitor of NFκB pathway (Gibcus et al. 2009).
(III) Immune evasion. The recurrent alterations of the chromosome region 9p24.1 in HRS cells result in the elevated expression of PD-1 ligand and are responsible for immune evasion of these neoplastic cells (Green et al. 2010). As the negative regulation of *PD-L1* expression by mir-138 was previously described in colorectal cancer, we hypothesize that downregulation of this potentially suppressive microRNA in cHL may constitute another HRS cells protective mechanism allowing to evade the host immune response (Zhao et al. 2016). Similarly, the oncogenic mir-9 can potentially contribute to the immune-evasion of HRS cells. This microRNA was shown to indirectly regulate the expression of several cytokines (IL-5, IL-6, CCL-5, TNF-alpha) that in turn may alter the immune infiltration in the tumor microenvironment (Leucci et al. 2012).

In summary, deregulated microRNAs can cooperate with canonical genetic changes to promote HRS formation and survival by involvement in B cell development, NFκB pathway, immune evasion, and possibly many other processes. In contrast to genetic alterations, microRNA expression can be easily modified by delivering mimics or inhibitors to the cells that makes microRNAs promising therapeutic targets that however require further investigation.

## Supplementary information

ESM 1 **Supplementary Table 1:** Detailed comparison between microRNA profiling studies. **Supplementary Table 2:** MicroRNAs referred to the Venn diagrams in Fig. 2. (XLSX 15.2 kb)

## Data Availability

Not applicable.
